# Apirhabdus apintestini gen. nov., sp. nov., a member of a novel genus of the family Enterobacteriaceae, isolated from the gut of the western honey bee Apis mellifera

**DOI:** 10.1099/ijsem.0.006346

**Published:** 2024-04-23

**Authors:** Matthew W. Quinn, Brendan A. Daisley, Sarah J. Vancuren, Amira Bouchema, Elina Niño, Gregor Reid, Graham J. Thompson, Emma Allen-Vercoe

**Affiliations:** 1 Department of Molecular and Cellular Biology, University of Guelph, Guelph, ON, N1G 2W1, Canada; 2 Department of Biology, Western University, London, ON, N6A 5C1, Canada; 3 Department of Entomology and Nematology, University of California, Davis, CA, 95616, USA; 4 University of California Agriculture and Natural Resources, Oakland, CA, 95618, USA; 5 Department of Microbiology & Immunology, Western University, London, ON, N6A 5B7, Canada

**Keywords:** *Apirhabdus apintestini*, *Apis mellifera*, CA-0114^T^, *Enterobacteriaceae*

## Abstract

A Gram-negative, motile, rod-shaped bacterial strain, CA-0114^T^, was isolated from the midgut of a western honey bee, *Apis mellifera*. The isolate exhibited ≤96.43 % 16S rRNA gene sequence identity (1540 bp) to members of the families *Enterobacteriaceae* and *Erwiniaceae*. Phylogenetic trees based on genome blast distance phylogeny and concatenated protein sequences encoded by conserved genes *atpD*, *fusA, gyrB*, *infB*, *leuS*, *pyrG* and *rpoB* separated the isolate from other genera forming a distinct lineage in the *Enterobacteriaceae*. In both trees, the closest relatives were *Tenebrionicola larvae* YMB-R21^T^ and *Tenebrionibacter intestinalis* BIT-L3^T^, which were isolated previously from *Tenebrio molitor* L., a plastic-eating mealworm. Digital DNA–DNA hybridization, orthologous average nucleotide identity and average amino acid identity values between strain CA-0114^T^ and the closest related members within the *Enterobacteriaceae* were ≤23.1, 75.45 and 76.04 %, respectively. The complete genome of strain CA-0114^T^ was 4 451669 bp with a G+C content of 52.12 mol%. Notably, the apparent inability of strain CA-0114^T^ to ferment d-glucose, inositol and l-rhamnose in the API 20E system is unique among closely related members of the *Enterobacteriaceae*. Based on the results obtained through genotypic and phenotypic analysis, we propose that strain CA-0114^T^ represents a novel species and genus within the family *Enterobacteriaceae*, for which we propose the name *Apirhabdus apintestini* gen. nov., sp. nov. (type strain CA-0114^T^=ATCC TSD-396^T^=DSM 116385^T^).

**Impact Statement**

The research presented here provides a significant insight into the phenotypic and genotypic characteristics of a novel bacterial species and genus belonging to the family *Enterobacteriaceae*. This work adds to the current literature by expanding the repertoire of known and characterized honey bee gut associated bacteria. Understanding the diversity and ecological roles of bacteria in the honeybee gut is crucial for honeybee health, which, in turn, has significant implications for pollination and agriculture.

## Introduction

The family *Enterobacteriaceae* consists of Gram-negative free-living or host-associated bacteria that can be isolated from a wide range of environments. In 2016, the *Enterobacteriaceae* faced taxonomic amendments when whole genome phylogeny was used to propose the order *Enterobacterales* and subdivide it into seven distinct families, namely *Budviciaceae*, *Enterobacteriaceae*, *Erwiniaceae*, *Hafniaceae*, *Morganellaceae*, *Pectobacteriaceae* and *Yersiniaceae* [1]. Since then, the family *Enterobacteriaceae* has undergone further taxonomic revision [2,5] and currently consists of 37 genera according to the List of Prokaryotic Names with Standing in Nomenclature [6]. Despite some exceptions, most members of the family are motile, catalase positive and oxidase negative. Members of the *Enterobacteriaceae* can benefit the host [7], but many are significant plant [8,9], human [10,11], animal [12,13] and insect [14,15] pathogens. Notably, certain members can cause serious illnesses in humans, including urinary tract infections [16,17], sepsis [18,19] and meningitis [20,21]. In the broader environment, some pathogenic members of the *Enterobacteriaceae* have reportedly been vectored by a variety of insects [3,9, 22, 23].

The western honey bee, *Apis mellifera*, is an important pollinator that contributes to crop security [24]. In recent years, the honey bee gut microbiota has been acknowledged as an important element that affects host health [25,27]. For example, core gut bacterial phylotypes (i.e., *Bartonella*, *Bifidobacterium*, Firm4, Firm5, *Gilliamella*, *Lactobacillus* and *Snodgrassella*) can contribute to bee fitness, nutrition and physiology through behavioural regulation [28], pathogen exclusion [29], immune system modulation [30], short-chain fatty acid production [31] and carbohydrate digestion [32]. However, members of the family *Enterobacteriaceae* are often associated with gut dysbiosis in honey bees [33] an d studies involving deep sequencing of bee gut bacterial communities have reported erratic distributions of *Enterobacteriaceae* species [34,35]. Moreover, members of the family are often associated with hive materials, flowers within the pollination environment and the honey crops of foraging bees exhibiting lower proportions of core gut microbiota members [33,36, 37].

This suggests that the *Enterobacteriaceae*, in general, represent environmental opportunists as opposed to being core members of the honey bee gut microbiota. The *Enterobacteriaceae* are also common symbionts of the honey bee parasitic mite *Varroa destructor* and are usually detected at higher levels in bees experiencing *Varroa* mite infestation or pesticide exposure [38,42]. Moreover, the ability of *Enterobacteriaceae* members to cause poor health outcomes in honey bees has been demonstrated previously. For example, *Escherichia coli* can significantly increase gut permeability, impair learning and memory ability, and shorten the lifespan of honey bees [43]. Likewise, *Klebsiella pneumoniae* and *Klebsiella aerogenes* are associated with bee dysbiosis and high mortality in larvae and adult bees [44]. In this study, we characterized strain CA-0114^T^, a representative isolate of the *Enterobacteriaceae* using phylogenetic and biochemical analysis. We propose that strain CA-0114^T^ represents a novel genus *Apirhabdus* with the description of *Apirhabdus apintestini* gen. nov., sp. nov. within the family *Enterobacteriaceae*.

## Isolation and ecology

Bacterial strain CA-0114^T^ was isolated on August 31st 2022 from the midgut homogenate of a western honey bee worker derived from a managed colony in Davis, California, USA (UC Davis apiaries). Briefly, 10 adult honey bees (stored at −20 °C following field collection) were thawed at 4 °C, surface sterilized with 70 % ethanol, and then rinsed with sterile ddH_2_O. Midguts were dissected from each of the samples, pooled together in 1 m sterile 0.01 M PBS, and then homogenized using a disposable plastic pestle (Fisher Scientific, catalogue number: 12-141-368). Subsequently, the homogenized suspension was serially diluted, plated on Columbia blood agar (CBA) supplemented with 5 % defibrinated sheep’s blood (cat. no. DSB500, Hemostat Laboratories) and incubated in an aerobic 5 % CO_2_ environment at 37 °C. One of many colonies appearing after 3 days of incubation (representing strain CA-0114^T^) was re-streaked three times on fresh CBA to obtain a pure culture and then preserved in a 20 % (v/v) glycerol solution at −80 °C.

## Genomic features and phylogeny

Genomic DNA of the isolate was prepared from freshly cultured biomass on CBA using the Qiagen QIAmp Power Faecal Pro DNA Kit as per manufacturer’s instructions. After DNA extraction and purification, the purity and concentration of genomic DNA was measured using Nanodrop and Qubit analysis. Long-read Nanopore sequencing was conducted by ligation using the SQK-LSK109 protocol and native barcoding with EXP-NBD104 on a MinION FLO-MIN106D flow cell. Raw Nanopore signals were basecalled and demultiplexed with the fast model of guppy version 6.3.8 with a minimum quality score of 8. Quality control and visualization of long-read sequencing data was performed using Nanoplot (https://github.com/wdecoster/NanoPlot) and adaptors were removed via Porechop version 0.2.4 (https://github.com/rrwick/Porechop) followed by quality filtering with Nanofilt (https://github.com/wdecoster/nanofilt) [45]. Long-read assembly (*de novo*) was conducted with Flye version 2.8.1-b1676 [46] with default parameters and the estimated genome size set to 4.5 MB. The trimmed reads were assembled into four circularized contigs with a combined length of 4 619 030 bp, mean coverage of 387 and an N50 value of 4 451 669. Strain CA-0114^T^ consisted of a circular chromosome ~4.4 MB long with a G+C content of 52.12 mol% and three extrachromosomal elements with sizes ranging from 86.8 to 36.4 kb. In the main chromosome, five prophage regions in addition to six incomplete prophage elements were predicted using the phaster (PHAge Search Tool Enhanced Release, default settings) webserver (http://phaster.ca/) [47]. Another two incomplete prophage regions were found in the plasmids. In the main chromosome, Prokka version 1.14.6 [48] annotation identified a total of 6164 genes and 6063 protein coding sequences with 2111 unique protein annotations (average protein length 193 aa). Further genomic predictions included 22 rRNA genes, 78 tRNA genes and four CRISPR repeat regions collectively harbouring 47 spacer sequences. Between the three plasmids, there were 243 protein coding sequences with 43 unique protein annotations. A genome plot of the main chromosome was constructed using DNA Plotter software Artemis 18.2.0 [49] to visualize the nucleotide positions of the genomic features (Fig. S1, available in the online version of this article). Raw fastq files were deposited to NCBI-SRA (SRR26893846) and the genome assembly was submitted to the DDBJ/EMBL/GenBank database under the BioProject accession number PRJNA973628.

**Fig. 1.**
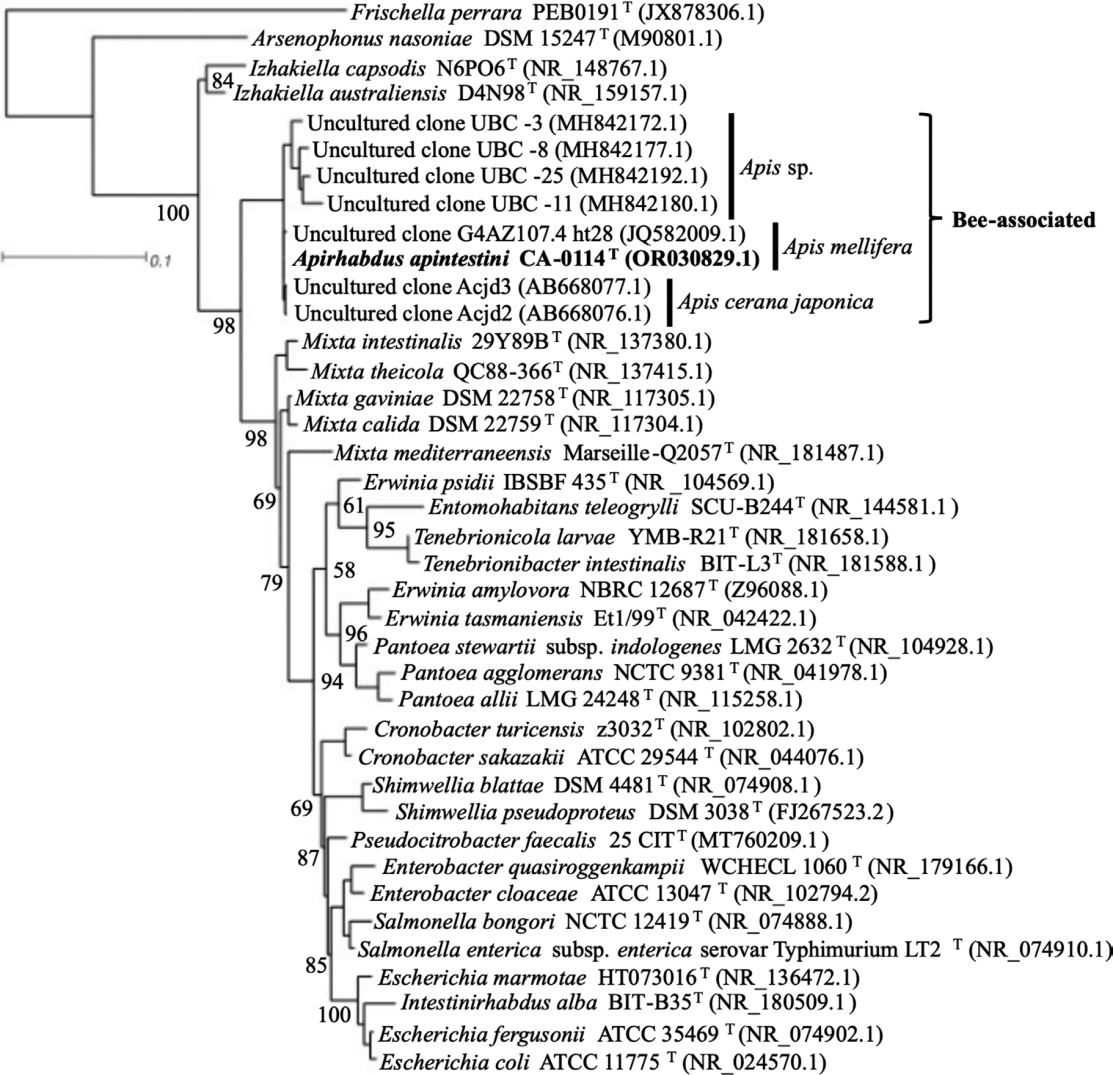
16S rRNA gene maximum likelihood phylogeny (1507 sites) of strain CA-0114^T^ and close relatives within the families *Enterobacteriaceae* and *Erwiniaceae* reconstructed using the Tamura–Nei substitution model (TN+F+I+G4). Previously uncultured bacterial clones are included and the names of the hosts of origin (for bee derived sequences) are highlighted. *Frischella perrara* PEB0191 is the outgroup. Bootstrap percentages calculated with 1000 replicates are indicated at nodes. Bar, 0.1 substations per nucleotide position.

To determine taxonomic relatedness, we performed comparative sequence analysis based on genome-derived 16S rRNA gene sequences between strain CA-0114^T^ and other described type species of the *Enterobacteriaceae* and *Erwiniaceae*. In the main chromosome of strain CA-0114^T^, seven 16AS rRNA genes copies were identified all having an average gene sequence identity of 99.96 % as determined via Clustal Omega (www.ebi.ac.uk/Tools/msa/clustalo/) [50]. The genome-derived consensus 16S rRNA sequence (1540 bp) of strain CA-0114^T^ that aligned best with the other six sequences was queried via blast against the NCBI databases limited to sequences from type material. The 16S rRNA gene had high sequence identity to sequences of taxa within the families *Enterobacteriaceae* and *Erwiniaceae* with the highest sequence match (96.43 % identity with 99 % query coverage) to a validly published species attributed to *Mixta gaviniae* DSM 22758^T^ (CP026377.1). Next, we performed maximum-likelihood tree reconstruction based on aligned 16S rRNA gene sequences of strain CA-0114^T^ and relevant type species of the *Enterobacteriaceae* and *Erwiniaceae* as well as seven honey bee associated uncultured clones. The 16S rRNA gene sequences of 38 related taxa were retrieved from NCBI and were aligned with the sequence of strain CA-0114^T^ using muscle [51]. The maximum-likelihood phylogeny was calculated via iq-tree version 1.6.12 [52] using the Tamura–Nei substitution model with four discrete gamma categories and invariable sites (TN+F+I+G4) as computed using ModelFinder [53]. The tree was visualized using Dendroscope version 3.8.5110 [54]. The honey bee associated bacterium *Frischella perrara* PEB0191 of the distantly related order *Orbales* was used to root the tree (1507 nucleotide sites). The 16S rRNA phylogeny (Fig. 1) placed strain CA-0114^T^ in the order *Enterobacterales* clustered within a distinct monophyletic honey bee clade that harboured seven uncultured bacterial clones, all previously associated with honey bees (*Apis*).

**Fig. 2.**
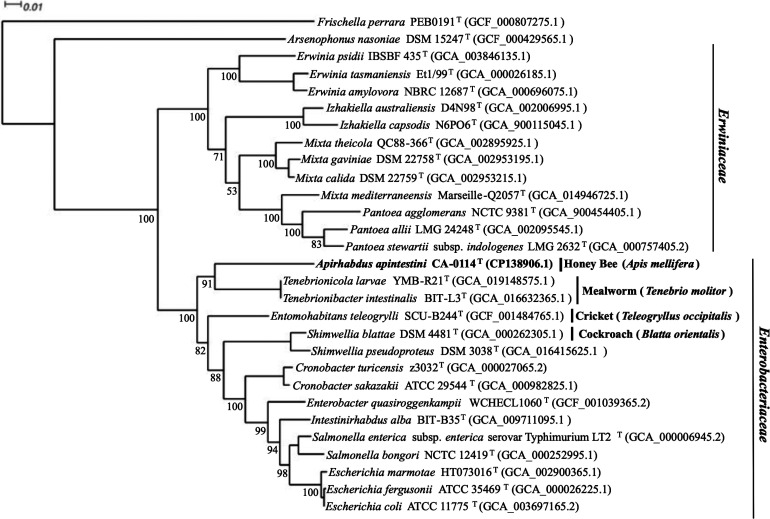
Conserved protein phylogeny for strain CA-0114^T^ and select type species of the families *Erwiniaceae* and *Enterobacteriaceae*. Hosts of origin for invertebrate-derived bacteria are highlighted. Maximum-likelihood tree reconstruction using the general matrix substitution model (LG+F+I+G4) was based on concatenated and aligned amino acids sequences (5300 total residues) from seven conserved proteins encoded by housekeeping genes *atpD*, *fusA*, *gyrB*, *infB*, *leuS, pyrG* and *rpoB* derived from 29 genomes. *Frischella perrara* PEB0191 is the outgroup. Ultrafast bootstrap support percentages (1000 replicates) are indicated at nodes. Bar, 0.01 substitutions per site.

**Fig. 3.**
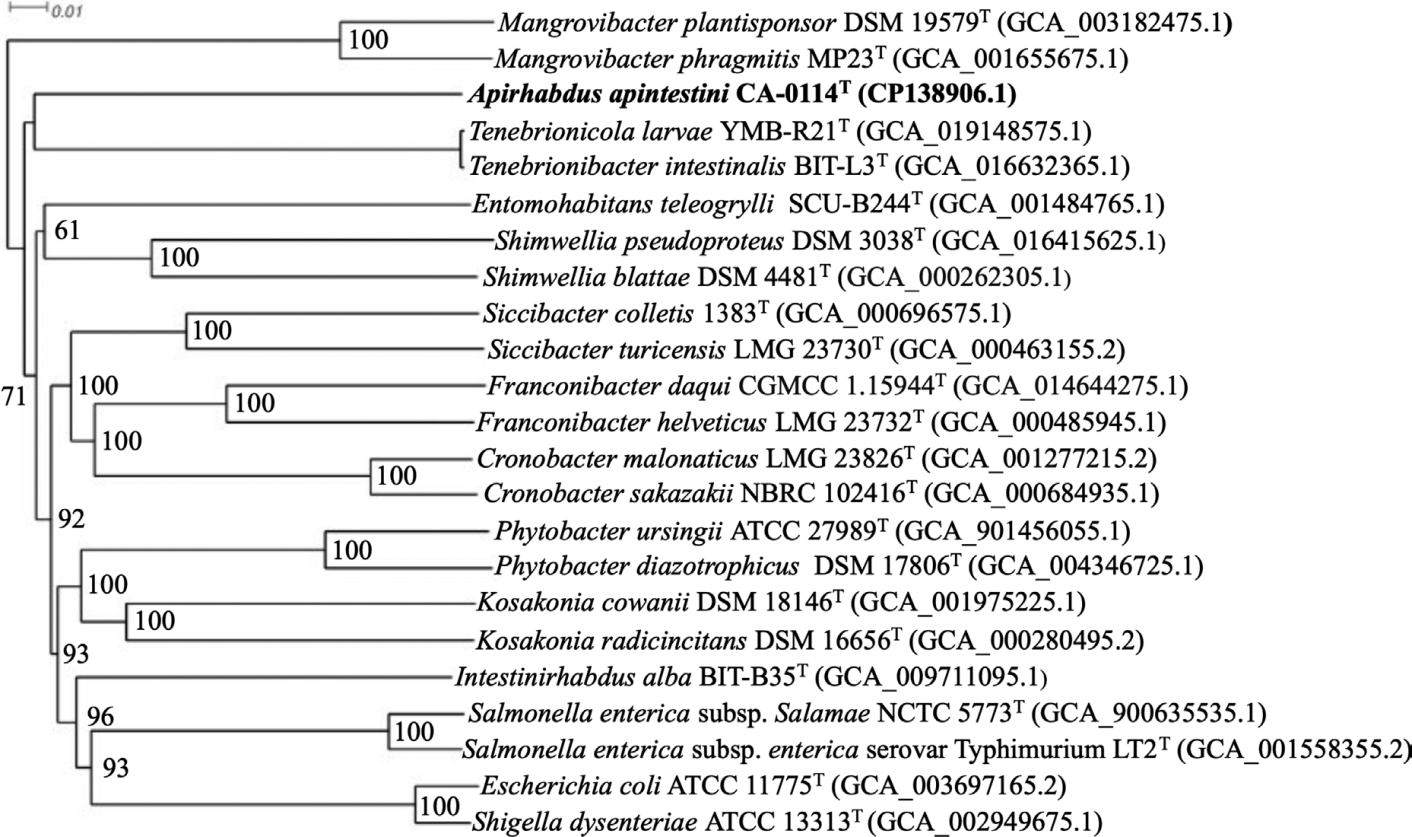
Balanced minimum evolution phylogenomic tree of strain CA-0114^T^ and closely related type strains within the family *Enterobacteriaceae*. The tree was reconstructed from genome pairwise comparisons conducted through the Type (Strain) Genome Server (https://tygs.dsmz.de/). Branch support was inferred from 100 pseudo-bootstrap replicates with values >60 % shown above nodes (average branch support of 92.3 %). The tree was rooted at the midpoint. Bar, 0.01 substitutions per site.

To obtain better taxonomic resolution, the phylogenetic position of strain CA-0114^T^ was inferred using multilocus sequence analysis (MLSA) and genome-based phylogenies. The MLSA tree was reconstructed based on the concatenated multiple sequence alignments of seven proteins encoded by conserved genes *atpD*, *fusA, gyrB*, *infB*, *leuS*, *pyrG* and *rpoB* (5300 residues) obtained from 28 publicly available *Erwiniaceae* and *Enterobacteriaceae* genomes using OrthoFinder version 2.5.4 [55]. muscle [51] was used to generate the multiple sequence alignment and maximum-likelihood tree reconstruction was performed via iq-tree 1.6.12 [52] using the general matrix substitution model with empirical codon frequencies, gamma-distributed rates and invariant sites (LG+F+I+G4) according to ModelFinder [53]. A second tree based on GBDP distances calculated from whole genome sequences of strain CA-0114^T^ and other related genera within the *Enterobacteriaceae* was reconstructed using the 'trimming' algorithm and distance formula d5 [56] available from the Type (Strain) Genome Server (https://tygs.dsmz.de/) [57] with branch support via FastME 2.1.6.1 [58]. All trees were visualized with Dendroscope version 3.8.5110 [54]. Conserved protein phylogeny (Fig. 2) allowed for separation of two distinct clades representing the *Enterobacteriaceae* and *Erwiniaceae*. Moreover, each genus formed a monophyletic group of its own with high bootstrap support. Specifically, strain CA-0114^T^ was monophyletic within the *Enterobacteriaceae*, and is sister group to genera *Tenebrionicola* and *Tenebrionibacter*. In the whole-genome based tree (Fig. 3), the formation of a distinct clade by strain CA-0114^T^ within the *Enterobacteriaceae* was confirmed. The clustering of the isolate within an unnamed monophyly supported the placement of strain CA-0114^T^ into a novel species of a novel genus within the family *Enterobacteriaceae*.

Calculations of average nucleotide identity (ANI) values were performed using the orthologous average nucleotide identity (OrthoANIu) calculator with default parameters [59] (www.ezbiocloud.net/tools/ani) and digital DNA–DNA hybridization (dDDH) values (Generalized Linear Model based) were calculated with the Genome-to-Genome Distance Calculator version 3.0 (https://ggdc.dsmz.de/ggdc.php) using formula 2 (identities/high-scoring pairs length) [56,60]. Average amino acid identity (AAI) values were determined using the Kostas lab AAI calculator (default settings) [61] (http://enve-omics.ce.gatech.edu/aai/). Strain CA-0114^T^ had higher AAI and OrthoANIu to members of the *Enterobacteriaceae* compared to members of the *Erwiniaceae* (Table 1). Moreover, values of 21.9 % (dDDH) and 75.45 % (OrthoANIu) were calculated between strain CA-0114^T^ and the closest related type strain *Tenebrionicola larvae* YMB-R21^T^, which are far below the species thresholds (70 and 95 % respectively) [62,63]. These results support the branching of strain CA-0114^T^ into a new species in the family *Enterobacteriaceae*. To clarify whether strain CA-0114^T^ could be assigned to a novel genus, the AAI values with the close phylogenetic neighbours were compared. AAI values between related bacteria that differ taxonomically at the genus level vary between ~60–80 % [64]. However, there is currently no universal AAI cut-off value that has been accepted for differentiating genera within the *Enterobacteriaceae*. We found that the highest AAI values for strain CA-0114^T^ were with *Tenebrionicola larvae* YMB-R21^T^ and *Tenebrionibacter intestinalis* BIT-L3^T^ at 76.04% and 75.64 %, respectively. These values closely match what is calculated between *Tenebrionicola larvae* YMB-R21^T^ and other genera within the *Enterobacteriaceae*. For example, *Tenebrionicola larvae* YMB-R21^T^ shared AAI values of 76.28% and 75.87% with *Cronobacter sakazakii* ATCC 29544^T^ and *Cronobacter turicensis* z3032 ^T^, respectively. Together, the conserved protein and whole genome phylogenies along with the OrthoANIu, AAI and dDDH results suggest that strain CA-0114^T^ represents a novel species belonging to a novel genus within the *Enterobacteriaceae*. The taxonomic novelty of strain CA-0114^T^ at the genus level within the *Enterobacteriaceae* was also confirmed using the GTDB taxonomic classifier version 1.7.0 software [65] (topology and ANI method) as implemented in the KBase server [66]. Interestingly, we found that the AAI, OrthoANIu, dDDH and 16S rRNA identity values between *Tenebrionibacter intestinalis* BIT-L3^T^ and *Tenebrionicola larvae* YMB-R21^T^ were alarmingly high (99.64, 99.83, 98.20 and 99.24 %, respectively) which suggests that these species are subjective synonyms [67,68].

**Table 1.** Comparison of OrthoANIu, AAI, and dDDH values as well as conserved protein and 16S rRNA gene sequence identity between strain CA-0114^T^ and closely related species within the *Enterobacteriaceae* and *Erwiniaceae*

**Species**	Type strain no.	Accessions used to generate table	OrthoANIu (%)	AAI (%)	dDDH [C.I.](%)	Conserved protein sequence identity* (%)	16S rRNA gene sequence identity [query coverage] (%)
***Enterobacteriaceae***							
*Tenebrionicola larvae*	YMB-R21^T^	GCA_019148575.1NR_181658.1	75.45	76.04	21.90 [19.6–24.3]	92.41	96.37 [96]
*Tenebrionibacter intestinalis*	BIT-L3^T^	GCA_016632365.1NR_181588.1	75.37	75.64	21.80 [19.5–24.2]	92.41	95.85 [93]
*Cronobacter sakazakii*	ATCC 29544^T^	GCA_000982825.1CP011047.1	74.83	73.88	20.90 [18.7–23.4]	91.82	95.61 [100]
*Cronobacter turicensis*	z3032^T^	GCA_000027065.2NR_102802.1	74.79	73.84	20.80 [18.6–23.2]	91.77	95.35 [100]
*Entomohabitans teleogrylli*	SCU B244^T^	GCA_001484765.1NR_144582.1	74.46	72.63	20.90 [18.7–23.4]	92.18	94.54 [91]
*Intestinirhabdus alba*	*BIT-B35* ^T^	GCA_009711095.1NR_180509.1	74.44	72.94	23.10 [20.8–25.5]	91.82	95.55 [91]
*Salmonellaenterica* subsp. e*nterica*	LT2^T^	GCA_000006945.2AE006468.2	74.30	72.61	21.40 [19.1–23.8]	91.93	95.79 [99]
*Shimwellia blattae*	DSM 4481^T^	GCA_000262305.1CP001560.1	74.27	73.26	21.80 [19.5–24.2]	91.29	95.02 [99]
*Enterobacter quasiroggenkampii*	WCHECL1060^T^	GCA_001039365.2NR_179166.1	74.14	73.46	20.60 [18.4–23]	91.56	95.65 [99]
***Erwiniaceae***							
*Mixta gaviniae*	DSM 22758^T^	GCA_002953195.1CP026377.1	73.48	69.16	21.80 [19.5–24.2]	89.60	96.43 [99]
*Mixta theicola*	QC88-366^T^	GCA_002895925.1NR_137415.1	73.39	69.19	20.00 [17.8–22.4]	89.55	96.26 [96]
*Mixta calida*	DSM 22759^T^	GCA_002953215.1CP026378.1	73.35	69.33	21.80 [19.5–24.2]	89.81	96.38 [100]
*Izhakiella australiensis*	D4N98^T^	GCA_002006995.1NR_159157.1	72.44	67.78	21.60 [19.4–24.1]	89.03	96.23 [99]
*Mixta mediterraneensis*	Marseille-Q2057^T^	GCA_014946725.1NR_181487.1	72.38	68.23	31.60 [29.2–34.1]	88.96	96.04 [99]
*Erwinia tasmaniensis*	Et1/99^T^	GCA_000026185.1CU468135.1	72.36	67.81	22.00 [19.8–24.5]	88.47	95.73 [100]
*Pantoea stewartii* subsp i*ndologenes*	LMG 2632^T^	GCA_000757405.2NR_104928.1	71.82	67.86	20.40 [18.2–22.8]	88.82	95.54 [98]
*Izhakiella capsodis*	N6PO6^T^	GCA_900115045.1NR_148767.1	71.42	68.16	19.50 [17.4–21.9]	88.58	95.68 [97]

a. *Conserved protein sequence identity refers to the amino acid sequence identity of the seven concatenated proteins (5300 amino acids) encoded by housekeeping genes (*atpD*, *fusA, gyrB*, *infB*, *leuS, pyrG, rpoB*) between strain CA-0114T and close relatives.

Genome functional assessment of strain CA-0114^T^ was performed with the Kyoto Encyclopedia of Genes and Genomes Database using the BlastKOALA web tool (prokaryotic database) (www.kegg.jp/blastkoala/) [69]. The analysis revealed a similar repertoire of metabolic genes comparable to *Tenebrionicola larvae* YMB-R21^T^ and *Tenebrionibacter intestinalis* BIT-L3^T^ (Table S1). For example, strain CA-0114^T^ contained genes encoding proteins involved in d-ribose, formate and mannitol utilization. However, unlike close relatives *Tenebrionicola larvae* YMB-R21^T^ and *Tenebrionibacter intestinalis* BIT-L3^T^, the isolate lacked genes involved in inositol, l-rhamnose and proline utilization and phosphotransferase system genes involved in the uptake of fructose and *N*-acetylmuramic acid. Strain CA-0114^T^ also showed a unique profile of ATP-binding cassette (ABC) transporter genes distinct from closely related type strains (Table S2). The isolate selectively contained the genes coding for ABC transporters specific to glutamine, glycine betaine/proline and microcin C, whereas the phylogenetically close relatives, *Tenebrionicola larvae* YMB-R21^T^ and *Tenebrionibacter intestinalis* BIT-L3^T^, lacked these but distinctively comprised the genes encoding the ABC transporter proteins related to branched-chain amino acid, erythritol, glutathione and iron-siderophore transport.

The genome of the isolate also encoded systems for flagella and fimbriae construction including transcriptional regulators, biosynthesis/assembly proteins, secretion chaperones, hook, ring, basal-body rod and associated proteins, motor switch proteins filament proteins, stator proteins as well as several genes associated with chemotaxis (Table S3). The discovery of flagellar genes was expected given the observed motility of strain CA-0114^T^ in semi solid media and motility was demonstrated previously [68] in the close relatives *Tenebrionicola larvae* YMB-R21^T^ and *Entomohabitans teleogrylli* KCTC 42022^T^ with both possessing genes for flagella biosynthesis and assembly. The genome also contained genes involved in colanic acid production which were absent in the genomes of the relatives *Tenebrionicola larvae* YMB-R21^T^, *Tenebrionibacter intestinalis* BIT-L3^T^, *Entomohabitans teleogrylli* SCU B244^T^ and *Shimwellia blattae* DSM 4481^T^. Among the extrachromosomal DNA, we notably found genes associated with type IV pili biogenesis.

Within the genome of strain CA-0114^T^ we found needle, secretin, export apparatus and ATPase genes involved in assembly and functioning of a type III secretion system which were absent in close relatives *Tenebrionicola larvae* YMB-R21^T^, *Tenebrionibacter intestinalis* BIT-L3^T^, *Entomohabitans teleogrylli* SCU B244^T^, *Shimwellia blattae* DSM 4481^T^ and *Cronobacter sakazakii* ATCC 29544^T^ (Table S3). Numerous pathogenic *Enterobacteriaceae* are reported to express type III secretion systems on their cell surface [70]; however, strain CA-0114^T^ lacked the complete set of type III secretion system genes found in the genome of *Salmonella enterica* subsp. *enterica* LT2^T^ [71]. Anti-SMASH predicted four genomic regions within the main chromosome associated with secondary metabolite synthesis. The region with the highest similarity to a known biosynthetic gene cluster (BGC) was a siderophore cluster that had high gene similarity to an aerobactin BGC from *Pantoea ananatis* [72]. The aerobactin siderophore has been identified previously as a virulence factor in entomopathogenic bacteria and is a key determinate in facilitating host association for plant pathogen strains of *Pantoea* [73,74]. Aerobactin is also required by avian pathogenic *Escherichia coli* strains, and human pathogen *Klebsiella pneumoniae* for virulence [75,76]. Genes for the biosynthesis of aerobactin were not found in the genomes of the close relative *Tenebrionicola larvae* YMB-R21^T^, *Tenebrionibacter intestinalis* BIT-L3^T^, *Shimwellia blattae* DSM 4481^T^ and *Salmonella enterica* subsp. *enterica* LT2^T^ (Table S3). These findings suggest a potential opportunistic role of strain CA-0114^T^ in the honey bee host; future investigations are warranted to determine if these genes are functionally expressed *in vivo*.

## Physiology and growth characteristics

Colony morphology and Gram stain of strain CA-0114^T^ was observed after 48 h of aerobic growth on CBA at 37 °C with 5 % CO_2_ . Cell morphology was observed using transmission electron microscopy (TEM). An on-drop negative stain method was used to prepare TEM grids. Briefly, 1 % uranyl acetate (UA) and wash buffer (20 mM HEPES pH 6.5) were filter sterilized with 0.2 µm syringe filters. Carbon-coated 100 copper mesh grids were glow discharged to remove charge effects and render the surface hydrophilic. The bacterial culture was grown in brain heart infusion (BHI) overnight and was used to inoculate Ringer’s medium at a 1 : 5 ratio. The resulting suspension was incubated at 37 °C for 4 h after which 10 µl was applied to the grid for 1 min followed by blotting with Whatman No. 1 filter paper. In a similar manner, the grid was immediately washed twice in the prepared buffer, briefly stained in 1 % UA followed immediate blotting and a final stain in 1 % UA for 20 s, and a final wash in sterile microbiology grade water. Images were acquired in an FEI Tecnai G2 F20 TEM (University of Guelph Electron Microscopy Facility). Motility was tested by stab inoculating fresh CA-0114^T^ into a semi solid medium prepared with 0.4 % agar, 0.3 % beef extract, 1 % pancreatic digest of casein and 0.5 % NaCl (https://asm.org/Protocols/Motility-Test-Medium-Protocol). The cultures were incubated at 37 °C with 5 % CO_2_ for 48 h. Swarming assays were performed as previously described [77]. Briefly, the swarming motility plates were prepared with 0.5 % agar, 1.0 % tryptone, 0.5 yeast extract and 0.5 % NaCl followed by supplementation with 0.5 % d-glucose after autoclave sterilization. Prior to inoculation with 2.5 µl overnight culture in BHI broth with yeast extract (BHI +YE), plates were dried at room temperature. Plates were then observed for swarming phenotype after a 24 h incubation at 37 °C with 5 % CO_2_.

Growth under anaerobic conditions was tested by incubating the strain for 4–5 days in an anaerobic chamber at 37 °C. Aerobic growth in BHI+YE was tested at different temperatures (23, 25, 33, 35, 37, 38, 39, 40 and 42 °C) and at multiple pH values using a disodium phosphate buffer (pH 7.0, 6.0, 5.5 and 5.0). Salt tolerance was determined by incubating the strain in BHI+YE medium at 37 °C supplemented with varying concentrations of NaCl (1, 4, 8 and 12 % w/v). For all conditions, growth was monitored with a spectrophotometer for 48 h set to double orbital shake (freq. 3 mm) with 600 nm absorbance measurements every 30 min. Oxidase activity was tested using BD BBL DrySlide Oxidase test slides and catalase activity was determined through release of oxygen from hydrogen peroxide on a glass slide. Antibiotic susceptibility was assessed by inoculating the strain on CBA in triplicate with antibiotic discs. The concentrations of each antibiotic were as follows; ceftriaxone (30 µg disc^−1^), ciprofloxacin (5 µg disc^−1^), enrofloxacin (5 µg disc^−1^) gentamicin (10 µg disc^−1^), lincomycin (2 µg disc^−1^), neomycin (30 µg disc^−1^), piperacillin (100 µg disc^−1^), sulphamethoxazole/trimethoprim (25 µg disc^−1^) and sulphonamides (300 µg disc^−1^). The presence of a zone of inhibition was used as a qualitative indication of susceptibility. Nitrate reduction was tested using nitrate reduction media prepared with 0.3 % (w/v) beef extract, 0.5 % (w/v) peptone and 0.1 % (w/v) KNO_3_ in 1000 ml deionized water. Enzyme and biochemical activities were recorded following 2 days incubation at 37 °C using the API 20E system (bioMérieux) according to the manufacturer’s instructions.

**Fig. 4.**
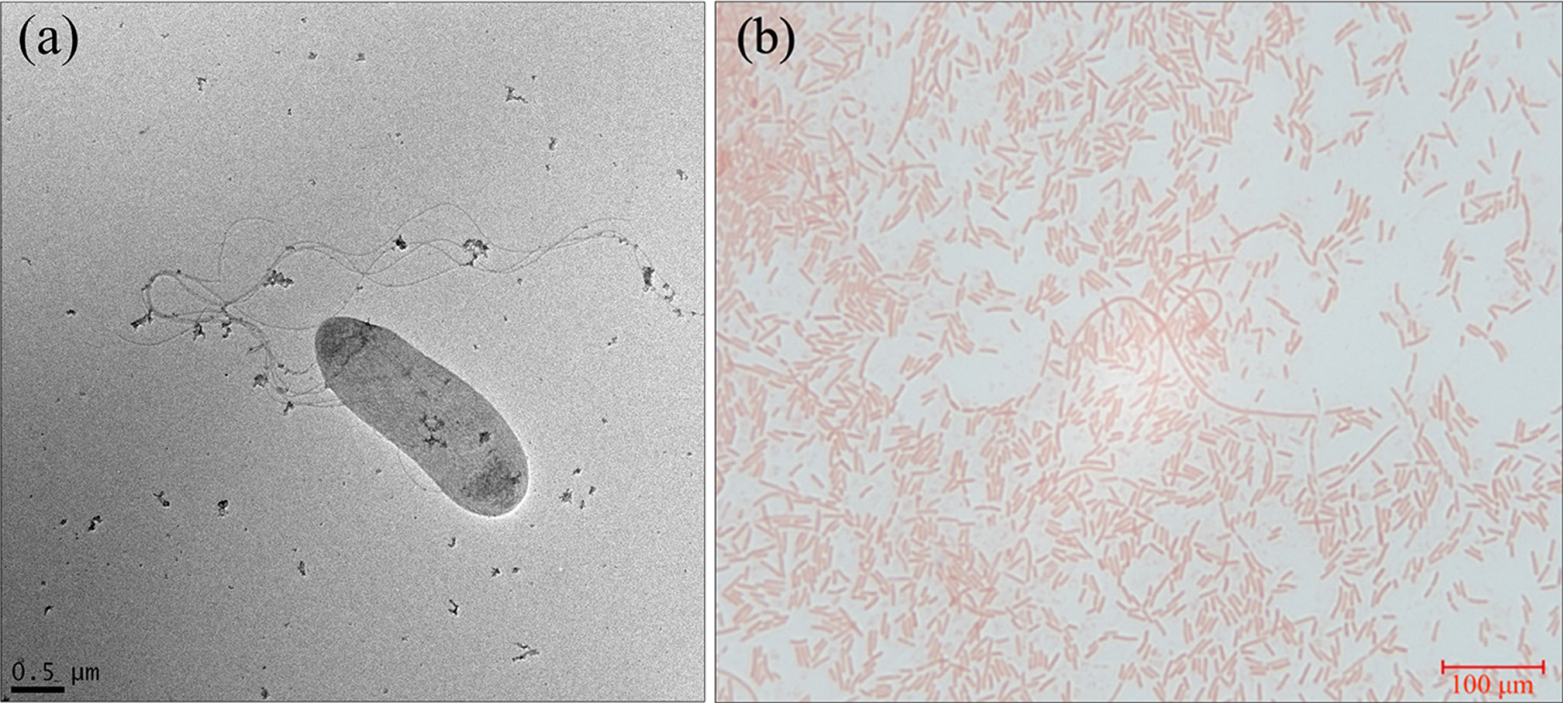
Transmission electron microscopy (TEM) and light microscopy of *A. apintestini* CA-0114^T^ cells. Images of TEM (**a**) and light microscope Gram stain reaction (**b**) are shown. The TEM image was taken on a FEI Tecnai G2 F20 and the Gram stain image was captured using a Zeiss AXIO Scope A1 Trinocular Pathology Microscope at ×1000 magnification using Zen Blue software.

Selection of optimal growth media was guided by previous recommendations for the study and characterization of honey bee gut microbes [78]. Strain CA-0114^T^ grew aerobically with 5 % CO_2_ on trypticase soy agar, colombia agar, CBA, MacConkey agar (producing pink colonies), fastidious anaerobe agar, fastidious anaerobe blood agar, brucella blood agar, BHI agar, and M17 agar but exhibited poor or no growth on Mueller–Hinton agar and malt extract–yeast extract–glucose–peptone agar. Strain CA-0114^T^ exhibited poor growth in M9 minimal media supplemented with d-fructose, d-glucose or sucrose as a sole carbon source. No haemolytic activity was observed when incubated on media containing 5 % sheep blood. Weak growth was observed on CBA after 4–5 days in anaerobic conditions (5 % CO_2_, 5 % H2, 90 % N_2_). Colonies of strain CA-0114^T^ were small (1–2 mm), circular, opaque with a glistening texture, convex elevation, grey colour and entire margins when grown aerobically on CBA. The bacterium was Gram-negative and transmission electron microscopy (TEM) revealed a rod-shaped bacterium approximately 1.8 µm long and 0.8 µm across with some cells expressing one to many long and thin flagella up to 12 µm long (Fig. 4). Motility, with the absence of swarming, was confirmed using semi solid media. Growth of strain CA-0114^T^ was observed at 23–39 °C (optimally at 37 °C), at pH 5.5–7.0 (optimally at pH 7.0) and was present with up to 1 % (w/v) NaCl, 12 % (w/v) d-fructose and 1 µg ml^−1^ tetracycline. Strain CA-0114^T^ was susceptible to ampicillin, ceftriaxone, ciprofloxacin, enrofloxacin, gentamicin, neomycin, piperacillin, sulphamethoxazole/trimethoprim and tetracycline but was resistant to lincomycin and sulphonamides. In the context of the API 20E system, strain CA-0114^T^ shared a similar enzymatic profile to those of *Tenebrionicola larvae* YMB-R21^T^ and *Tenebrionibacter intestinalis* BIT-L3^T^ (Table 2). However, strain CA-0114^T^ was uniquely unable to reduce nitrate to nitrite and lacked the full set of genes involved in dissimilatory nitrate reduction unlike closely related members of the *Enterobacteriaceae* (Table S4). The isolate also differed from *Tenebrionicola larvae* YMB-R21^T^ and *Tenebrionibacter intestinalis* BIT-L3^T^ in its inability to ferment d-glucose, inositol and l-rhamnose. The traits of strain CA-0114^T^ are summarized in the species description.

**Table 2.** Differential biochemical characteristics between strain CA-0114^T^ and closely related members within the family *Enterobacteriaceae* Strains: 1, CA-0114^T^; 2, *Tenebrionicola larvae* YMB-R21^T^; 3, *Tenebrionibacter intestinalis* BIT-L3^T^. Data for *Tenebrionicola larvae* YMB-R21^T^ (GCA_019148575.1) was obtained from Lee *et al*. [68]. Data for *Tenebrionibacter intestinalis* BIT-L3^T^ (GCA_016632365.1) was obtained from Hu and Yang [67]. +, Positive result; −, negative result; nd, no data; /+, expected positive result based on genome sequences; /−, expected negative result based on genome sequences.

Characteristic	1	2	3
Reaction/enzyme:			
Arginine dihydrolase	−	−	−
β-Galactosidase	−/−	−/−	−/−
Lysine decarboxylase	−/−	nd/−	−/−
Ornithine decarboxylase	−/−	nd/−	−/−
Citrate utilization	−/−	−/−	−/−
H_2_S production	−	nd	−
Urease	-/-	+/+	-/+
Tryptophan deaminase	−	nd	−
Indole	−/−	−/−	−/−
Acetoin production	−	nd	−
Gelatinase	−/−	−/−	−/−
Oxidase	−/−	+/−	−/−
Catalase	+/+	−/+	+/+
Reduce NO_3_^-^ to NO_2_^-^	−/−	+/+	nd/+
Fermentation/oxidation of:			
d-Glucose	−	+	+
d-Mannitol	+/+	+/+	+/+
Inositol	−/−	+/+	+/+
d-Sorbitol	−	−	−
l-Rhamnose	−/−	+/+	+/+
Sucrose	−	nd	−
Melibiose	−	−	−
Amygdalin	−	nd	−
l-Arabinose	−	−	nd

Therefore, based on genome relatedness, distinct biochemical traits as well as phylogeny, strain CA-0114^T^ represents a novel species and genus within the family *Enterobacteriaceae*, for which the name *Apirhabdus apintestini* gen. nov., sp. nov. is proposed. We also recommend taxonomic revaluation of *Tenebrionibacter intestinalis* BIT-L3^T^ and *Tenebrionicola larvae* YMB-R21^T^ based on their high genomic relatedness.

## Description of *Apirhabdus* gen. nov.

*Apirhabdus* (A.pi.rhab’dus. L. fem. n. *apis* bee; Gr. fem. n. *rhabdos*, rod; N.L. fem. n. *Apirhabdus*, a rod from a bee).

A member of the family *Enterobacteriaceae*. Cells are Gram-reaction-negative, rapidly motile by means of peritrichous flagella, mesophilic, chemoorganotrophic, facultatively anaerobic rods (approx. 1.8×0.8 µm) that appear singly or in chains. Oxidase activity is negative, catalase activity is positive and urease activity is negative. Growth occurs at 23–39 °C, at pH as low as 5.5, at 0–1 % (w/v) NaCl and in the presence of 0–12 % (w/v) d-fructose. Nitrate is not reduced. The G+C content is 52.1 mol%. Members of this genus are expected to form a branch distinct from other genera in phylogenetic trees based on concatenated amino acid sequences encoded by housekeeping genes *atpD*, *fusA*, *gyrB*, *infB*, *leuS*, *pyrG* and *rpoB*. The type species is *Apirhabdus apintestini*.

## Description of *Apirhabdus apintestini* sp. nov

*Apirhabdus apintestini* (ap.in.tes.ti’ni. L. fem. n. *apis*, a bee; L. neut. n. *intestinum*, gut; N.L. gen. n. *apintestini*, of the intestine of a bee).

In addition to characteristics given in the genus description, growth occurs on trypticase soy agar, Columbia agar and CBA, but poor growth is observed on Mueller–Hinton agar and malt extract–yeast extract–glucose–peptone agar. Colonies grown on CBA are circular (1–2 mm), opaque with a glistening texture, convex elevation, grey colour and entire margins within 48 h at 37 °C (5 % CO_2_). Optimal growth occurs at 37 °C and pH 7.5 in BHI+YE. Swarming motility and haemolytic activity are absent. β-Galactosidase, citrate utilization, gelatinase, arginine dihydrolase, lysine decarboxylase, ornithine decarboxylase and tryptophan deaminase are negative. Unable to ferment d-glucose, inositol and l-rhamnose in the API 20E system.

The type strain, CA-0114^T^ (=ATCC TSD-396^T^=DSM 116385^T^), was isolated from the gut of western honey bee (*A. mellifera*) from California, USA. The DNA G+C content of the type strain is 52.1 mol%. The GenBank/EMBL/DDBL accession numbers for the whole genome and 16S rRNA gene sequence data of strain CA-0114^T^ are CP138906 and OR030829, respectively.

## supplementary material

10.1099/ijsem.0.006346 Uncited Supplementary Material 1.

## Abbreviations

AAI: average amino acid identity
ABC: ATP-binding cassette
BGC: biosynthetic gene cluster
BHI+YE: brain heart infusion broth with yeast extract
CBA: Columbia blood agar
dDDH: digital DNA–DNA hybridization
GBDP: genome blast distance phylogeny
LB: lysogeny broth
MLSA: multilocus sequence analysis
OrthoANIu: orthologous average nucleotide identity
UA: uranyl acetate
