# Supplementary material for: Apirhabdus apintestini gen. nov., sp. nov., a member of a novel genus of the family Enterobacteriaceae, isolated from the gut of the western honey bee Apis mellifera
Source: Int J Syst Evol Microbiol. 2024 Apr 23;74(4):006346. doi: 10.1099/ijsem.0.006346 (PMC11092227; doi:10.1099/ijsem.0.006346)
Supplement: Uncited Supplementary Material 1. [file ijsem-74-06346-s001.pdf]

Supplementary information

Apirhabdus apintestini gen. nov., sp. nov., a member of a novel genus of the family *Enterobacteriaceae*, isolated from the gut of the western honeybee *Apis mellifera*.

Matthew W. Quinn[1], Brendan A. Daisley[1,2], Sarah Vancuren[1], Amira Bouchema[1], Elina Niño[3,4], Gregor Reid[5], Graham J. Thompson[2], Emma Allen-Vercoe[1,#].

[1]Department of Molecular and Cellular Biology, University of Guelph, Guelph, ON, N1G 2W1, Canada. [2]Department of Biology, Western University, London, ON, N6A 5C1, Canada. [3]Department of Entomology and Nematology, University of California, Davis, Davis, CA, 95616, United States. [4]University of California Agriculture and Natural Resources, Oakland, CA, 95618, United States. [5]Department of Microbiology & Immunology, Western University, London, ON, N6A 5B7, Canada.

[#]Correspondence to: Dr. Emma Allen-Vercoe, (eav@uoguelph.ca)

This supplementary information document contains:

- Supplementary Figure 1 and Tables 1-4

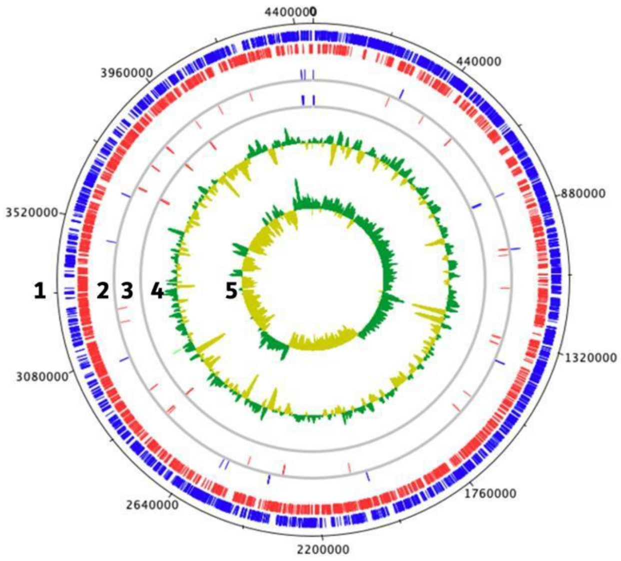

Figure S1. Genomic DNA plot for strain CA-0114^T. Circles with plus strand (clockwise in blue) and minus strand (anticlockwise in red) represent (moving towards the center) the nucleotide position of: 1, coding DNA (bp); 2, tRNA genes; 3, rRNA genes; 4, fraction of G+C bases within a sliding window of 10-kb, plotted between the minimum and maximum values (green and yellow indicate values > and < the average G+C content, respectively); 5, GC skew ([G - C]/[G + C] plotted with a 10-kb window between the min and max values (green indicates a bias toward G and yellow indicates a bias towards C).

Table S1. Metabolic genes selectively present in the genomes of strain CA-0114^T and closely related type species.

Strains: 1, CA-0114^T (PRJNA973628); 2, *Tenebrionicola larvae* YMB-R21^T (GCA_019148575.1); 3, *Tenebrionibacter intestinalis* BIT-L3^T (GCA_016632365.1); 4, *Entomohabitans teleogrylli* SCU B244^T (GCA_001484765.1); 5, *Shimwellia blattae* DSM 4481^T (GCA_000262305.1) 6, *Cronobacter sakazakii* ATCC 29544^T (GCA_000982825.1); 7, *Salmonella* enterica subsp. *enterica* LT2^T (GCA_000006945.2). +, present; -, absent.

Genes	KEGG Protein Orthology	1	2	3	4	5	6	7
**Amino acid metabolism**								
dat; D-alanine transaminase (EC:2.6.1.21)	K00824	-	-	-	+	+	-	-
putA; RHH-type transcriptional regulator, proline utilization regulon repressor / proline dehydrogenase / delta 1-pyrroline-5-carboxylate dehydrogenase [EC:1.5.5.2 1.2.1.88]	K13821	-	+	+	+	+	+	+
**Carbohydrate metabolism**								
**L-Arabinose**								
araA; L-arabinose isomerase (EC:5.3.1.4)	K01804	+	-	-	+	+	+	+
araB; L-ribulokinase (EC:2.7.1.16)	K00853	-	-	-	+	+	+	+
araC; AraC family transcriptional regulator, arabinose operon regulatory protein	K02099	-	-	-	+	+	+	+
araD, *ulaF*, *sgaE*, *sgbE*; L-ribulose-5-phosphate 4-epimerase (EC:5.1.3.4)	K03077	-	-	-	+	+	+	+
**Citrate**								
citC; [citrate (pro-3S)-lyase] ligase (EC:6.2.1.22)	K01910	-	-	-	+	+	+	+
citD; citrate lyase subunit gamma (acyl carrier protein)	K01646	-	-	-	+	+	+	+
citE; citrate lyase subunit beta / citryl-CoA lyase (EC:4.1.3.34)	K01644	-	-	-	+	+	+	+
citF; citrate lyase subunit alpha / citrate CoA-transferase (EC:2.8.3.10)	K01643	-	-	-	+	+	+	+
citG; triphosphoribosyl-dephospho-CoA synthase (EC:2.4.2.52)	K05966	-	-	-	+	+	+	+
citX; holo-ACP synthase (EC:2.7.7.61)	K05964	-	-	-	+	+	+	+
dpiA, *citB*; two-component system, CitB family, response regulator CitB	K07702	-	-	-	+	+	+	+
dpiB, citA; two-component system, CitB family, cit operon sensor histidine kinase CitA (EC:2.7.13.3)	K07700	-	-	-	+	+	+	+
**Formate**								
fdnI; formate dehydrogenase-N, gamma subunit	K08350	+	+	+	+	+	+	+
fdnH; formate dehydrogenase-N, beta subunit	K08349	+	+	+	+	+	+	+
fdnG; formate dehydrogenase-N, alpha subunit [EC:1.17.5.3]	K08348	+	+	+	+	+	+	+
**Inositol**								
iolB; 5-deoxy-glucuronate isomerase (EC:5.3.1.30)	K03337	-	+	+	-	-	+	+

iolC; 5-dehydro-2-deoxygluconokinase (EC:2.7.1.92)	K03338	-	+	+	-	-	+	+
iolD; 3D-(3,5/4)-trihydroxycyclohexane-1,2-dione acylhydrolase (decyclizing) (EC:3.7.1.22)	K03336	-	+	+	-	-	+	+
iolE; inosose dehydratase (EC:4.2.1.44)	K03335	-	+	+	-	-	+	+
iolG; myo-inositol 2-dehydrogenase / D-chiro-inositol 1-dehydrogenase (EC:1.1.1.18 1.1.1.369)	K00010	-	+	+	-	-	+	+
iolH; myo-inositol catabolism protein IolH	K06605	-	+	+	-	-	+	+
iolI; 2-keto-myo-inositol isomerase (EC:5.3.99.11)	K06606	-	+	+	-	-	+	+
iolT; MFS transporter, SP family, major inositol transporter	K06609	-	+	+	-	-	+	-
mmsA, *iolA*, ALDH6A1; malonate-semialdehyde dehydrogenase (acetylating) / methylmalonate-semialdehyde dehydrogenase (EC:1.2.1.18 1.2.1.27)	K00140	-	+	+	-	-	+	+
**L-lactate**								
lldD; L-lactate dehydrogenase (cytochrome) (EC:1.1.2.3)	K00101	-	-	-	+	+	+	+
lldE; L-lactate dehydrogenase complex protein LldE	K18928	+	+	+	-	+	-	-
lldF; L-lactate dehydrogenase complex protein LldF	K18929	+	+	+	-	+	-	-
lldG; L-lactate dehydrogenase complex protein LldG	K00782	-	+	+	-	+	-	-
lldP, *lctP*; L-lactate permease	K00427	+	+	+	+	+	+	+
lldR; GntR family transcriptional regulator, L-lactate dehydrogenase operon regulator	K14348	-	-	-	-	+	+	+
**Lactose**								
lacY; MFS transporter, OHS family, lactose permease	K02532	-	-	-	-	+	+	-
lacZ; beta-galactosidase (EC:3.2.1.23)	K01190	-	-	-	+	-	+	-
**L-rhamnose**								
rhaA; L-rhamnose isomerase (EC:5.3.1.14)	K01813	+	+	+	+	+	+	+
rhaB; rhamnulokinase (EC:2.7.1.5)	K00848	+	+	+	+	+	+	+
rhaD; rhamnulose-1-phosphate aldolase (EC:4.1.2.19)	K01629	+	+	+	+	+	+	+
rhaM; L-rhamnose mutarotase (EC:5.1.3.32)	K03534	+	+	+	+	+	+	+
rhaR; AraC family transcriptional regulator, L-rhamnose operon transcriptional activator RhaR	K02854	+	+	+	+	+	+	+
rhaS; AraC family transcriptional regulator, L-rhamnose operon regulatory protein RhaS	K02855	-	+	+	+	+	+	+
rhaT; L-rhamnose-H+ transport protein	K02856	+	+	+	+	+	+	+
**Mannitol**								
mtlD; mannitol-1-phosphate 5-dehydrogenase (EC:1.1.1.17)	K00009	+	+	+	+	-	+	+
mtlR; mannitol operon repressor	K02562	+	+	+	+	-	+	+
**Melibiose**								
melA; alpha-galactosidase (EC:3.2.1.22)	K07406	-	-	-	+	-	-	+
melB; melibiose permease	K11104	-	-	-	+	-	-	+
melR; AraC family transcriptional regulator, melibiose operon regulatory protein	K23237	-	-	-	+	-	-	+
**D-Ribose**								
rbsA; ribose transport system ATP-binding protein (EC:7.5.2.7)	K10441	+	+	+	+	+	+	+
rbsB; ribose transport system substrate-binding protein	K10439	+	+	+	+	+	+	+
rbsC; ribose transport system permease protein	K10440	+	+	+	+	+	+	+
rbsD; D-ribose pyranase (EC:5.4.99.62)	K06726	+	+	+	+	+	+	+
rbsK, RBKS; ribokinase (EC:2.7.1.15)	K00852	+	+	+	+	+	+	+
**Xylose**								

xylA; xylose isomerase (EC:5.3.1.5)	K01805	-	-	-	+	+	+	+
xylB, XYLB; xylulokinase (EC:2.7.1.17)	K00854	-	-	-	+	+	+	+
xylE; MFS transporter, SP family, xylose:H+ symportor	K08138	-	+	+	+	+	-	+
xylF; D-xylose transport system substrate-binding protein	K10543	-	-	-	-	-	+	-
xylG; D-xylose transport system ATP-binding protein (EC:7.5.2.10)	K10545	-	-	-	-	-	+	-
xylH; D-xylose transport system permease protein	K10544	-	-	-	-	-	+	-
xylS, *yicI*; alpha-D-xyloside xylohydrolase (EC:3.2.1.177)	K01811	-	-	-	-	-	+	-
**Phosphotransferase systems**								
**Ascorbate**								
ulaA, *sgaT*; ascorbate PTS system EIIC component	K03475	-	-	-	+	+	-	+
ulaB, *sgaB*; ascorbate PTS system EIIB component (EC:2.7.1.194)	K02822	-	-	-	+	+	-	+
ulaC, *sgaA*; ascorbate PTS system EIIA or EIIAB component (EC:2.7.1.194)	K02821	-	-	-	+	+	-	+
**Cellobiose**								
celA, *chbB*; cellobiose PTS system EIIB component (EC:2.7.1.196 2.7.1.205)	K02760	-	-	-	+	-	+	+
celB, *chbC*; cellobiose PTS system EIIC component	K02761	-	-	-	+	-	+	+
celC, *chbA*; cellobiose PTS system EIIA component (EC:2.7.1.196 2.7.1.205)	K02759	-	-	-	+	-	+	+
**Fructose**								
fruA; fructose PTS system EIIBC or EIIC component (EC:2.7.1.202)	K02770	-	+	+	+	+	+	+
fruB, fpr; multiphosphoryl transfer protein (EC:2.7.1.202)	K11183	+	+	+	+	+	+	+
fruK; 1-phosphofructokinase (EC:2.7.1.56)	K00882	+	+	+	+	+	+	+
**Glucitol/sorbitol**								
srlA; glucitol/sorbitol PTS system EIIC component	K02783	-	-	-	-	-	-	+
srlB; glucitol/sorbitol PTS system EIIA component (EC:2.7.1.198)	K02781	-	-	-	-	-	-	+
srlE; glucitol/sorbitol PTS system EIIB component (EC:2.7.1.198)	K02782	-	-	-	-	-	-	+
**Glucose**								
crr; sugar PTS system EIIA component (EC:2.7.1.-)	K02777	+	+	+	+	+	+	+
ptsG; glucose PTS system EIICB or EIICBA component (EC:2.7.1.199)	K02779	+	-	-	+	+	+	+
**Mannitol**								
mtlA, cmtA; mannitol PTS system EIICBA or EIICB component (EC:2.7.1.197)	K02800	+	+	+	+	-	+	+
**N-Acetylmuramic acid**								
murP; N-acetylmuramic acid PTS system EIICB component (EC:2.7.1.192)	K11192	-	+	+	-	-	-	-
**Sucrose**								
scrA, *sacP*, *sacX*, *ptsS*; sucrose PTS system EIIBCA or EIIBC component (EC:2.7.1.211)	K02810	+	+	+	-	+	+	+

Table S2. ABC transporter genes selectively present in the genomes of strain CA-0114^T and closely related type species

Strains: 1, CA-0114^T (PRJNA973628); 2, *Tenebrionicola larvae* YMB-R21^T (GCA_019148575.1); 3, *Tenebrionibacter intestinalis* BIT-L3^T (GCA_016632365.1); 4, *Entomohabitans teleogrylli* SCU B244^T (GCA_001484765.1); 5, *Shimwellia blattae* DSM 4481^T (GCA_000262305.1) 6, *Cronobacter sakazakii* ATCC 29544^T (GCA_000982825.1); 7, *Salmonella* enterica subsp. *enterica* LT2^T (GCA_000006945.2). +, present; -, absent.

Genes	KEGG Protein Orthology	1	2	3	4	5	6	7
**Alkanesulfonate**								
ssuA; sulfonate transport system substrate-binding protein	K15553	-	-	-	+	+	+	-
ssuB; sulfonate transport system ATP-binding protein (EC:7.6.2.14)	K15555	-	-	-	+	+	+	-
ssuC; sulfonate transport system permease protein	K15554	-	-	-	+	+	+	-
**Branched-chain amino acids**								
livF; branched-chain amino acid transport system ATP-binding protein	K01996	-	+	+	+	+	+	+
livG; branched-chain amino acid transport system ATP-binding protein	K01995	-	+	+	+	+	+	+
livH; branched-chain amino acid transport system permease protein	K01997	-	+	+	+	+	+	+
livK; branched-chain amino acid transport system substrate-binding protein	K01999	-	+	+	+	+	+	+
livM; branched-chain amino acid transport system permease protein	K01998	-	+	+	+	+	+	+
**Cobalt**								
cbiM; cobalt/nickel transport system permease protein	K02007	-	-	-	+	+	-	+
cbiN; cobalt/nickel transport protein	K02009	-	-	-	+	+	-	+
cbiO; cobalt/nickel transport system ATP-binding protein	K02006	-	-	-	+	+	-	+
cbiQ; cobalt/nickel transport system permease protein	K02008	-	-	-	+	+	-	+
**D-allose**								
alsA; D-allose transport system ATP-binding protein (EC:7.5.2.8)	K10551	-	-	-	-	+	-	-
alsB; D-allose transport system substrate-binding protein	K10549	-	-	-	-	+	-	-
alsC; D-allose transport system permease protein	K10550	-	-	-	-	+	-	-
**Erythritol**								
eryE; erythritol transport system ATP-binding protein	K17204	-	+	+	+	-	-	-
eryF; erythritol transport system permease protein	K17203	-	+	+	+	-	-	-
glpK, GK; glycerol kinase (EC:2.7.1.30)	K00864	-	+	+	+	-	-	-
**Galactofuranose**								
ytfQ; galactofuranose transport system substrate-binding protein	K23508	-	-	-	+	+	+	-

ytfR; galactofuranose transport system ATP-binding protein (EC:7.5.2.9)	K10820	-	-	-	+	+	+	-
ytfT, yjfF; galactofuranose transport system permease protein	K23509	-	-	-	+	+	+	-
**Glutamine**								
glnH; glutamine transport system substrate-binding protein	K10036	+	-	-	+	+	+	+
glnP; glutamine transport system permease protein	K10037	+	-	-	+	+	+	+
glnQ; glutamine transport system ATP-binding protein (EC:7.4.2.1)	K10038	+	-	-	+	+	+	+
**Glutathione**								
gsiA; glutathione transport system ATP-binding protein	K13892	-	+	+	+	+	+	+
gsiB; glutathione transport system substrate-binding protein	K13889	-	+	+	+	+	+	+
gsiC; glutathione transport system permease protein	K13890	-	+	+	+	+	+	+
gsiD; glutathione transport system permease protein	K13891	-	+	+	+	+	+	+
**Glycine betaine/proline**								
proV; glycine betaine/proline transport system ATP-binding protein (EC:7.6.2.9)	K02000	+	-	-	+	+	+	+
proW; glycine betaine/proline transport system permease protein	K02001	+	-	-	+	+	+	+
proX; glycine betaine/proline transport system substrate-binding protein	K02002	+	-	-	+	+	+	+
**Histidine**								
hisJ; histidine transport system substrate-binding protein	K10014	-	-	-	+	+	+	+
hisM; histidine transport system permease protein	K10015	-	-	-	+	+	+	+
hisP; histidine transport system ATP-binding protein (EC:7.4.2.1)	K10017	-	-	-	+	+	+	+
hisQ; histidine transport system permease protein	K10016	-	-	-	+	+	+	+
**HMP/FAMP**								
thiX; putative hydroxymethylpyrimidine transport system permease protein	K15599	-	-	-	-	+	-	-
thiY; putative hydroxymethylpyrimidine transport system substrate-binding protein	K15598	-	-	-	-	+	-	-
thiZ; putative hydroxymethylpyrimidine transport system ATP-binding protein	K15600	-	-	-	-	+	-	-
**Iron (II)/manganese**								
sitA; manganese/iron transport system substrate-binding protein	K11604	-	-	-	+	+	-	+
sitB; manganese/iron transport system ATP-binding protein	K11607	-	-	-	+	+	-	+
sitC; manganese/iron transport system permease protein	K11605	-	-	-	+	+	-	+
sitD; manganese/iron transport system permease protein	K11606	-	-	-	+	+	-	+
**Iron-siderophore**								
fepA, *pfeA*, *iroN*, *pirA*; ferric enterobactin receptor	K19611	-	+	+	+	+	+	+
fepB; ferric enterobactin transport system substrate-binding protein	K23185	-	+	+	+	+	+	+
fepC, *fagC*, *cchE*, *desF*; iron-siderophore transport system ATP-binding protein (EC:7.2.2.17 7.2.2.-)	K23188	-	+	+	+	+	+	+
fepD, *fagA*, *cchC*, *desH*; iron-siderophore transport system permease protein	K23186	-	+	+	+	+	+	+
fepG, *fagB*, *cchD*, *desG*; iron-siderophore transport system permease protein	K23187	-	+	+	+	+	+	+
**L-arabinose**								
araF; L-arabinose transport system substrate-binding protein	K10537	-	-	-	+	+	+	-
araG; L-arabinose transport system ATP-binding protein (EC:7.5.2.12)	K10539	-	-	-	+	+	+	-
araH; L-arabinose transport system permease protein	K10538	-	-	-	+	+	+	-
**Lysine/arginine/ornithine**								

argT; lysine/arginine/ornithine transport system substrate-binding protein	K10013	-	-	-	+	+	+	+
hisM; histidine transport system permease protein	K10015	-	-	-	+	+	+	+
hisP; histidine transport system ATP-binding protein (EC:7.4.2.1)	K10017	-	-	-	+	+	+	+
hisQ; histidine transport system permease protein	K10016	-	-	-	+	+	+	+
**Maltose/maltodextrin**								
malE; maltose/maltodextrin transport system substrate-binding protein	K10108	-	-	-	+	+	+	+
malF; maltose/maltodextrin transport system permease protein	K10109	-	-	-	+	+	+	+
malG; maltose/maltodextrin transport system permease protein	K10110	-	-	-	+	+	+	+
malK, *mtlK*, *thuK*; multiple sugar transport system ATP-binding protein (EC:7.5.2.-)	K10111	-	-	-	+	+	+	+
**Microcin C**								
yejA; microcin C transport system substrate-binding protein	K13893	+	-	-	+	+	+	+
yejB; microcin C transport system permease protein	K13894	+	-	-	+	+	+	+
yejE; microcin C transport system permease protein	K13895	+	-	-	+	+	+	+
yejF; microcin C transport system ATP-binding protein	K13896	+	-	-	+	+	+	+
**Molybdate**								
modA; molybdate transport system substrate-binding protein	K02020	+	+	+	+	+	+	+
modB; molybdate transport system permease protein	K02018	+	+	+	+	+	+	+
modC; molybdate transport system ATP-binding protein (EC:7.3.2.5)	K02017	-	+	+	+	+	+	+
modE; molybdate transport system regulatory protein	K02019	+	+	+	+	+	+	+
modF; molybdate transport system ATP-binding protein	K05776	-	+	+	+	+	+	+
**Nickel**								
nikA, *cntA*; nickel transport system substrate-binding protein	K15584	-	-	-	+	+	-	-
nikB, *cntB*; nickel transport system permease protein	K15585	-	-	-	+	+	-	-
nikC, *cntC*; nickel transport system permease protein	K15586	-	-	-	+	+	-	-
nikD, *cntD*; nickel transport system ATP-binding protein (EC:7.2.2.11)	K15587	-	-	-	-	+	-	-
nikE, *cntF*; nickel transport system ATP-binding protein (EC:7.2.2.11)	K10824	-	-	-	+	+	-	-
**Taurine**								
tauA; taurine transport system substrate-binding protein	K15551	-	-	-	+	+	+	-
tauB; taurine transport system ATP-binding protein (EC:7.6.2.7)	K10831	-	-	-	-	+	+	-
tauC; taurine transport system permease protein	K15552	-	-	-	+	+	+	-
**Urea**								
urtA; urea transport system substrate-binding protein	K11959	-	-	-	+	-	+	-
urtB; urea transport system permease protein	K11960	-	-	-	+	-	+	-
urtC; urea transport system permease protein	K11961	-	-	-	+	-	+	-
urtD; urea transport system ATP-binding protein	K11962	-	-	-	+	-	+	-
urtE; urea transport system ATP-binding protein	K11963	-	-	-	+	-	+	-

Table S3. Aerobactin, biofilm, flagellar and secretion system genes selectively present in the genomes of strain CA-0114^{T} and closely related type species

Strains: 1, CA-0114^{T} (PRJNA973628); 2, *Tenebrionicola larvae* YMB-R21^{T} (GCA_019148575.1); 3, *Tenebrionibacter intestinalis* BIT-L3^{T} (GCA_016632365.1); 4, *Entomohabitans teleogrylli* SCU B244^{T} (GCA_001484765.1); 5, *Shimwellia blattae* DSM 4481^{T} (GCA_000262305.1) 6, *Cronobacter sakazakii* ATCC 29544^{T} (GCA_000982825.1); 7, *Salmonella* enterica subsp. *enterica* LT2^{T} (GCA_000006945.2). +, Present; -, absent.

**Genes**	**KEGG Protein Orthology**	**1**	**2**	**3**	**4**	**5**	**6**	**7**
**Aerobactin biosynthesis (Lysine → Aerobactin)**								
iucA; N2-citryl-N6-acetyl-N6-hydroxylysine synthase (EC:6.3.2.38)	K03894	+	-	-	+	-	+	-
iucB; acetyl CoA:N6-hydroxylysine acetyl transferase (EC:2.3.1.102)	K03896	+	-	-	+	-	+	-
iucC; aerobactin synthase (EC:6.3.2.39)	K03895	+	-	-	+	-	+	-
iucD; lysine N6-hydroxylase (EC:1.14.13.59)	K03897	+	-	-	+	-	+	-
**Biofilm biosynthesis**								
**Cellulose biosynthesis**								
bcsA; cellulose synthase (UDP-forming) (EC:2.4.1.12)	K00694	-	+	+	+	+	+	+
bcsB; cellulose synthase operon protein B	K20541	-	+	+	+	+	+	+
bcsC; cellulose synthase operon protein C	K20543	-	+	+	+	+	+	+
**Colanic acid biosynthesis**								
wcaA; putative colanic acid biosynthesis glycosyltransferase WcaA	K25875	+	-	-	-	-	+	+
wcaB; putative colanic acid biosynthesis acetyltransferase WcaB (EC:2.3.1.-)	K03819	+	-	-	-	-	+	+
wcaC; putative colanic acid biosynthesis glycosyltransferase WcaC (EC:2.4.-.-)	K13684	+	-	-	-	-	+	+
wcaE; putative colanic acid biosynthesis glycosyltransferase WcaE (EC:2.4.-.-)	K13683	+	-	-	-	-	+	+
wcaF; putative colanic acid biosynthesis acetyltransferase WcaF (EC:2.3.1.-)	K03818	+	-	-	-	-	+	+
wcaI; putative colanic acid biosynthesis glycosyltransferase WcaI	K03208	+	-	-	-	-	+	+
wcaJ; undecaprenyl-phosphate glucose phosphotransferase (EC:2.7.8.31)	K03606	+	-	-	-	-	+	+
**Curli Fimbrae biosynthesis**								
csgA; major curlin subunit	K04334	-	-	-	-	+	-	+
csgB; minor curlin subunit	K04335	-	-	-	-	+	-	+
csgC; curli production protein	K04336	-	-	-	-	+	-	+
csgD; *LuxR* family transcriptional regulator, csgAB operon transcriptional regulatory protein	K04333	-	-	-	-	+	-	+
csgE; curli production assembly/transport component CsgE	K04337	-	-	-	-	+	-	+

csgF; curli production assembly/transport component CsgF	K04338	-	-	-	-	+	-	+
csgG; curli production assembly/transport component CsgG	K06214	-	-	-	-	+	-	+
**Gycogen biosynthesis**								
glgA; starch synthase (EC:2.4.1.21)	K00703	-	+	+	+	+	+	+
glgC; glucose-1-phosphate adenylyltransferase (EC:2.7.7.27)	K00975	-	+	+	+	+	+	+
glgP; glycogen phosphorylase (EC:2.4.1.1)	K00688	-	+	+	+	+	+	+
**Flagellar biosynthesis and assembly**								
cheY; Chemotaxis regulator CheY	K03413	-	+	+	+	-	+	+
fliH; Flagellar assembly protein FliH	K02411	+	+	+	+	-	+	+
fliL; Flagellar basal body-associated protein FliL	K02415	+	+	+	+	-	+	+
flgA; Flagellar basal-body P-ring formation protein FlgA	K02386	+	+	+	+	-	+	+
flgD; Flagellar basal-body rod modification protein FlgD	K02389	+	+	+	+	-	+	+
flgB; Flagellar basal-body rod protein FlgB	K02387	+	+	+	+	-	+	+
flgC; Flagellar basal-body rod protein FlgC	K02388	+	+	+	+	-	+	+
flgF; Flagellar basal-body rod protein FlgF	K02391	+	+	+	+	-	+	+
flgG; Flagellar basal-body rod protein FlgG	K02392	+	+	+	+	-	+	+
flgN; Flagellar biosynthesis protein FlgN	K02399	+	+	+	+	-	+	+
flhA; Flagellar biosynthesis protein FlhA	K02400	+	+	+	+	-	+	+
flhB; Flagellar biosynthesis protein FlhB	K02401	+	+	+	+	-	+	+
fliO; Flagellar biosynthesis protein FliO	K02418	+	+	+	+	-	+	+
fliP; Flagellar biosynthesis protein FliP	K02419	+	+	+	+	-	+	+
fliQ; Flagellar biosynthesis protein FliQ	K02420	+	+	+	+	-	+	+
fliR; Flagellar biosynthesis protein FliR	K02421	+	+	+	+	-	+	+
fliS; Flagellar biosynthesis protein FliS	K02422	+	+	+	+	-	+	+
fliD; Flagellar cap protein FliD	K02407	+	+	+	+	-	+	+
flgE; Flagellar hook protein FlgE	K02390	+	+	+	+	-	+	+
flgK; Flagellar hook-associated protein FlgK	K02396	+	+	+	+	-	+	+
flgL; Flagellar hook-associated protein FlgL	K02397	+	+	+	+	-	+	+
fliE; Flagellar hook-basal body complex protein FliE	K02408	+	+	+	+	-	+	+
fliK; Flagellar hook-length control protein FliK	K02414	-	-	-	+	-	+	+
flgH; Flagellar L-ring protein FlgH	K02393	+	+	+	+	-	+	+
fliF; Flagellar M-ring protein FliF	K02409	+	+	+	+	-	+	+
motA; Flagellar motor rotation protein MotA	K02556	+	+	+	+	-	+	+
motB; Flagellar motor rotation protein MotB	K02557	+	+	+	+	-	+	+
fliG; Flagellar motor switch protein FliG	K02410	+	+	+	+	-	+	+
fliM; Flagellar motor switch protein FliM	K02416	+	+	+	+	-	+	+
fliN; Flagellar motor switch protein FliN	K02417	+	+	+	+	-	+	+
flgI; Flagellar P-ring protein FlgI	K02394	+	+	+	+	-	+	+
flgJ; Flagellar protein FlgJ [peptidoglycan hydrolase]	K02395	+	+	+	+	-	+	+
flhE; Flagellar protein FlhE	K03516	+	+	+	+	-	+	+

fliJ; Flagellar protein FliJ	K02413	-	+	+	+	-	+	+
flhC; Flagellar transcriptional activator FlhC	K02402	+	+	+	+	-	+	+
flhD; Flagellar transcriptional activator FlhD	K02403	+	+	+	+	-	+	+
fliC; Flagellin FliC	K02406	+	+	+	+	-	+	+
fliI; Flagellum-specific ATP synthase FliI (EC:7.4.2.8)	K02412	+	+	+	+	-	+	+
fliA; RNA polymerase sigma factor FliA	K02405	+	+	+	+	-	+	+
**Secretion systems biosynthesis and assembly**								
**Type III secretion system**								
invE; type III secretion system protein	K22511	-	-	-	-	-	-	+
invG; type III secretion system outer membrane ring protein	K22504	-	-	-	-	-	-	+
prgH; type III secretion system protein	K22488	-	-	-	-	-	-	+
prgJ; type III secretion system protein	K22487	-	-	-	-	-	-	+
prgK; type III secretion system inner membrane ring protein	K22505	-	-	-	-	-	-	+
spaK, *invB*; type III secretion system chaperone	K22512	-	-	-	-	-	-	+
spaL; type III secretion system ATPase (EC:7.4.2.8)	K22506	-	-	-	-	-	-	+
spaM, *invI*; type III secretion system protein	K22513	-	-	-	-	-	-	+
spaN, *invJ*; type III secretion system protein	K22514	-	-	-	-	-	-	+
spaP; type III secretion system export apparatus protein	K22507	-	-	-	-	-	-	+
spaQ; type III secretion system export apparatus protein	K22508	+	-	-	-	-	-	+
spaR; type III secretion system export apparatus protein	K22509	-	-	-	-	-	-	+
spaS; type III secretion system export apparatus switch protein	K22510	-	-	-	-	-	-	+
sseK2; type III secretion system effector	K23945	-	-	-	-	-	-	+
yscC, *sctC*, *ssaC*; type III secretion protein C	K03219	+	-	-	-	-	-	+
yscD, *sctD*, *ssaD*; type III secretion protein D	K03220	-	-	-	-	-	-	+
yscF, *sctF*, *ssaG*, *prgI*; type III secretion protein F	K03221	+	-	-	-	-	-	+
yscJ, *sctJ*, *hrcJ*, *ssaJ*; type III secretion protein J	K03222	-	-	-	-	-	-	+
yscN, *sctN*, *hrcN*, *ssaN*; ATP synthase in type III secretion protein N (EC:7.4.2.8)	K03224	+	-	-	-	-	-	+
yscQ, *sctQ*, *hrcQ*, *ssaQ*, *spaO*; type III secretion protein Q	K03225	-	-	-	-	-	-	+
yscR, *sctR*, *hrcR*, *ssaR*; type III secretion protein R	K03226	-	-	-	-	-	-	+
yscS, *sctS*, *hrcS*, *ssaS*; type III secretion protein S	K03227	+	-	-	-	-	-	+
yscT, *sctT*, *hrcT*, *ssaT*; type III secretion protein T	K03228	-	-	-	-	-	-	+
yscU, *sctU*, *hrcU*, *ssaU*; type III secretion protein U	K03229	+	-	-	-	-	-	+
yscV, *sctV*, *hrcV*, *ssaV*, *invA*; type III secretion protein V	K03230	+	-	-	-	-	-	+
**Type VI secretion system**								
hcp; type VI secretion system secreted protein Hcp	K11903	+	+	+	+	+	+	+
impA; type VI secretion system protein ImpA	K11902	+	-	-	+	-	+	+
impB; type VI secretion system protein ImpB	K11901	+	+	+	+	+	+	+
impC; type VI secretion system protein ImpC	K11900	+	+	+	+	+	+	+
impE; type VI secretion system protein ImpE	K11898	+	-	-	+	-	+	+
impF; type VI secretion system protein ImpF	K11897	+	-	-	+	-	+	+

impG, *vasA*; type VI secretion system protein ImpG	K11896	+	+	+	+	+	+	+
impH, *vasB*; type VI secretion system protein ImpH	K11895	+	+	+	+	+	+	+
impJ, *vasE*; type VI secretion system protein ImpJ	K11893	+	+	+	+	+	+	+
impK, *ompA*, *vasF*, *dotU*; type VI secretion system protein ImpK	K11892	+	+	+	+	+	+	+
impL, *vasK*, *icmF*; type VI secretion system protein ImpL	K11891	+	+	+	+	+	+	+
impM; type VI secretion system protein ImpM	K11890	-	-	-	+	-	+	+
K11905; type VI secretion system protein	K11905	+	+	+	+	+	+	-
vgrG, Type VI secretion system secreted protein VgrG	K11904	+	+	+	+	+	+	+
vasD, *lip*; type VI secretion system protein VasD	K11906	+	+	+	+	+	+	+
vasG, *clpV*; type VI secretion system protein VasG	K11907	+	+	+	+	+	+	-
vasJ; type VI secretion system protein VasJ	K11910	+	+	+	+	+	+	-
vasL; type VI secretion system protein VasL	K11911	+	+	+	+	+	+	-

Table S4. Key enzymatic genes selectively present in the genomes of strain CA-0114^{T} and closely related type species

Strains: 1, CA-0114^{T} (PRJNA973628); 2, *Tenebrionicola larvae* YMB-R21^{T} (GCA_019148575.1); 3, *Tenebrionibacter intestinalis* BIT-L3^{T} (GCA_016632365.1); 4, *Entomohabitans teleogrylli* SCU B244^{T} (GCA_001484765.1); 5, *Shimwellia blattae* DSM 4481^{T} (GCA_000262305.1) 6, *Cronobacter sakazakii* ATCC 29544^{T} (GCA_000982825.1); 7, *Salmonella* enterica subsp. *enterica* LT2^{T} (GCA_000006945.2). +, present; -, absent.

**Genes**	**KEGG Protein Orthology**	**1**	**2**	**3**	**4**	**5**	**6**	**7**
**Catalase**								
katE, CAT, *catB*, *srpA*; catalase (EC:1.11.1.6)	K03781	+	+	+	+	+	+	+
**Dissimaltory nitrate reduction**								
napA; nitrate reductase (cytochrome) (EC:1.9.6.1)	K02567	-	+	+	+	+	-	+
napB; nitrate reductase (cytochrome), electron transfer subunit	K02568	-	+	+	+	+	-	+
narG, *narZ*, *nxrA*; nitrate reductase / nitrite oxidoreductase, alpha subunit (EC:1.7.5.1 1.7.99.-)	K00370	+	+	+	+	+	+	+
narH, *narY*, *nxrB*; nitrate reductase / nitrite oxidoreductase, beta subunit (EC:1.7.5.1 1.7.99.-)	K00371	+	+	+	+	+	+	+
narI, *narV*; nitrate reductase gamma subunit (EC:1.7.5.1 1.7.99.-)	K00374	+	+	+	+	+	+	+
nirB; nitrite reductase (NADH) large subunit (EC:1.7.1.15)	K00362	+	+	+	+	+	+	+
nirD; nitrite reductase (NADH) small subunit (EC:1.7.1.15)	K00363	+	+	+	+	+	+	+
nrfA; nitrite reductase (cytochrome c-552) (EC:1.7.2.2)	K03385	-	+	+	+	+	-	+
**Indole production**								
tnaA; tryptophanase (EC:4.1.99.1)	K01667	-	-	-	-	-	-	-
**Beta-galactosidase**								
lacZ; beta-galactosidase (EC:3.2.1.23)	K01190	-	-	-	+	-	+	-
**Lysine decarboxylase**								
ldcC, *cadA*; lysine decarboxylase (EC:2.6.1.21)	K01582	-	-	-	+	+	+	+
**Ornithine decarboxylase**								
speF; ornithine decarboxylase (EC:4.1.1.17)	K01581	-	-	-	+	+	+	+
**Urease**								
ureA; urease subunit gamma (EC:3.5.1.5)	K01430	-	+	+	-	-	-	-
ureB; urease subunit beta (EC:3.5.1.5)	K01429	+	+	+	-	-	-	-
ureC; urease subunit alpha (EC:3.5.1.5)	K01428	-	+	+	-	-	-	-
ureD, *ureH*; urease accessory protein	K03190	+	+	+	-	-	-	-

ureE; urease accessory protein	K03187	+	+	+	-	-	-	-
ureF; urease accessory protein	K03188	+	+	+	-	-	-	-
ureG; urease accessory protein	K03189	+	+	+	-	-	-	-
